# Enlarging Plexiform Tumor in a Pregnant Patient with Neurofibromatosis Type One

**DOI:** 10.7759/cureus.2802

**Published:** 2018-06-13

**Authors:** Stefanie Williams

**Affiliations:** 1 GME, Madigan Army Medical Center, University Place, USA

**Keywords:** plexiform neurofibroma, neurofibromatosis type 1 (nf1), malignant peripheral nerve sheath tumor

## Abstract

Neurofibromatosis type one (NF1) is a relatively common genetic disorder, however, it is rare to see in pregnancy. There are few case reports detailing a link between increasing tumor size in pregnant patients with NF1 likely due to the increase in hormones. However, some neurofibromas, like the plexiform, can undergo malignant degeneration into aggressive malignant peripheral nerve sheath tumors (MPNST). Any patient with NF1 should undergo a prompt evaluation and biopsy of a plexiform neurofibroma if it starts to change in size or consistency. Due to the increased risk of malignancy in NF1 patients and the poor survival rates in MPNST, it should never be assumed that tumors enlarging in pregnancy are due to hormones. The case below details the enlargement of a plexiform neurofibroma on a 21-year-old gravida two parity one female at 28 weeks with NF1.

## Introduction

Neurofibromatosis type one (NF1) is a multisystem disorder caused by a germline mutation in the NF gene on chromosome 17q11.2 that encodes the neurofibromin protein [1]. The loss of this key regulator in cell reproduction is responsible for the occurrence of benign and malignant tumors in this condition. NF1 occurs in about one in every 3500 people worldwide and shows no gender or ethnic predominance [1,2]. A de novo mutation occurs in about half of all people with this disorder while others inherit NF1 in an autosomal dominant pattern [2]. The most widely accepted diagnostic criteria for NF1 is the National Institute of Health (NIH) Consensus Criteria (Table 1). Clinical diagnosis of this disorder can be made with two of the seven findings. These physical findings can occur at different ages, which can make the diagnosis difficult if the patient does not have continuity of care.

**Table 1:** NIH Criteria for Neurofibromatosis Type One Diagnosis. NIH: National Institute of Health; NF1: Neurofibromatosis Type One.

NIH Criteria
Freckling of the axillary or inguinal region
Two or more neurofibromas or one plexiform neurofibromas
Two or more Lisch Nodules
Café au lait macules: Six or more must be present
Osseous lesion
Optic pathway glioma
First degree relative with NF1

**Figure 1:**
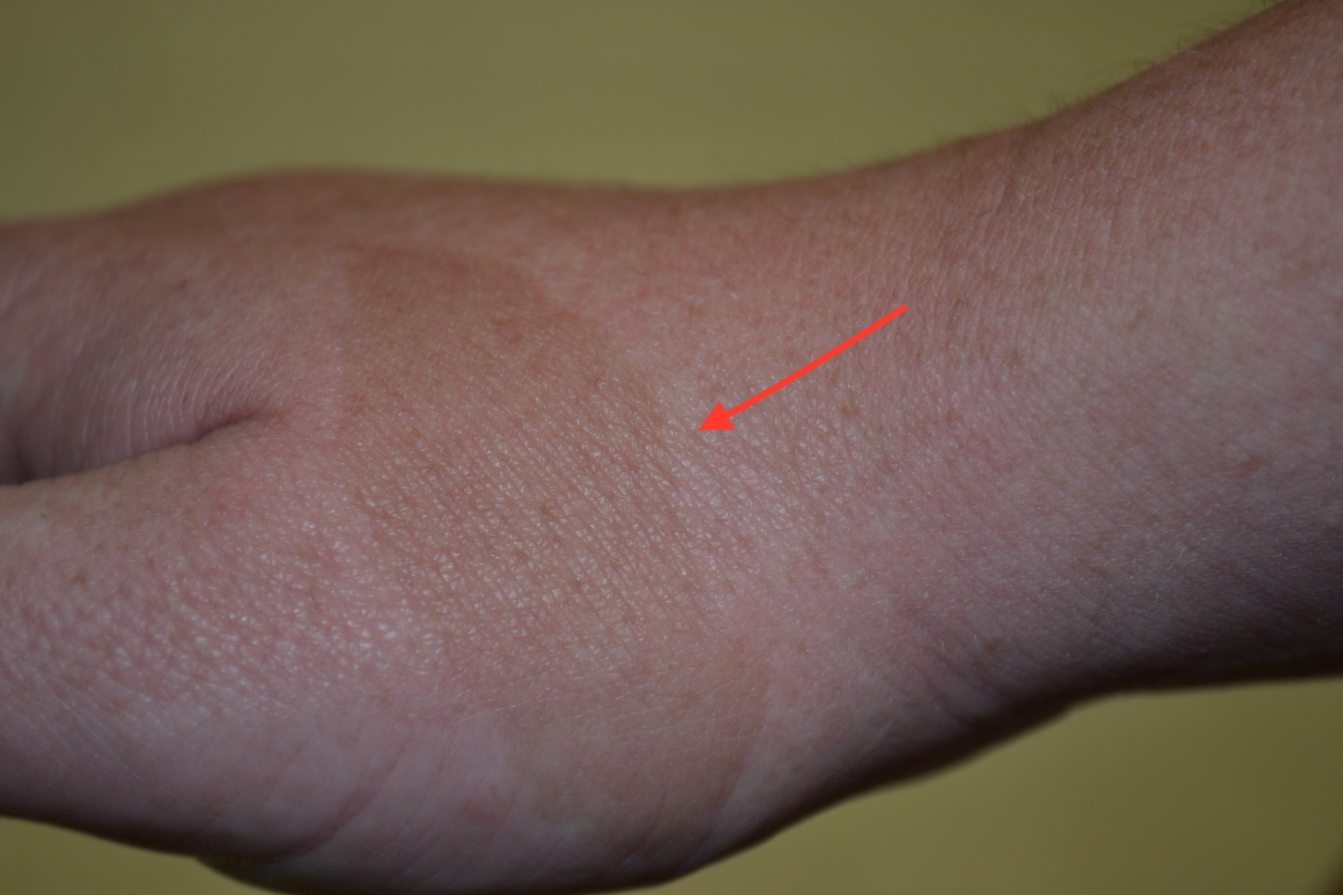
Café Au Lait Macule.

The first three criteria are hyperpigmentation abnormalities, which were under close inspection visible in the patient described below. The first of the seven criteria to emerge is the café au lait macule, which can be seen in the first two years of life (Figure 1). In order to meet criteria, there must be six or more with size greater than half a millimeter if prepubescent or greater than fifteen millimeters if postpubescent [1]. These macules are typically dark tan to brown with smooth borders.

Freckling in areas of skin apposition like the axilla or the inguinal region is another characteristic on NF1, which can be easily missed. The freckling typically does not appear until five to eight years of age and unlike solar-induced freckles these are typically in sun-protected areas [1]. Lisch nodules, which are benign hamartomas of the iris, are present in almost all individuals with NF1 although smaller ones may be hard to see without slit lamp [2].

Neurofibromas are the most striking of the diagnostic criteria and can occur in the hundreds to thousands on the skin of the person. There are four subtypes of neurofibromas that are seen in NF1 with the plexiform being the largest and most problematic. The other three subtypes include the cutaneous, subcutaneous, and spinal. The neurofibromas in the skin are the most readily visible and can occur in numbers ranging from hundreds to covering almost all visible skin [1]. These can be particularly debilitating to the patient as they can be painful, disrupt proper fit to clothing, and cause social isolation due to cosmetic concerns. The spinal neurofibromas can be problematic due to their impingement on nerve roots. Only two neurofibromas or one plexiform tumor is needed to meet the NIH criteria. The other criteria in the NIH guideline are: distinctive osseous lesion, optic pathway glioma, or a first-degree relative with NF1 [1].

## Case presentation

**Figure 2:**
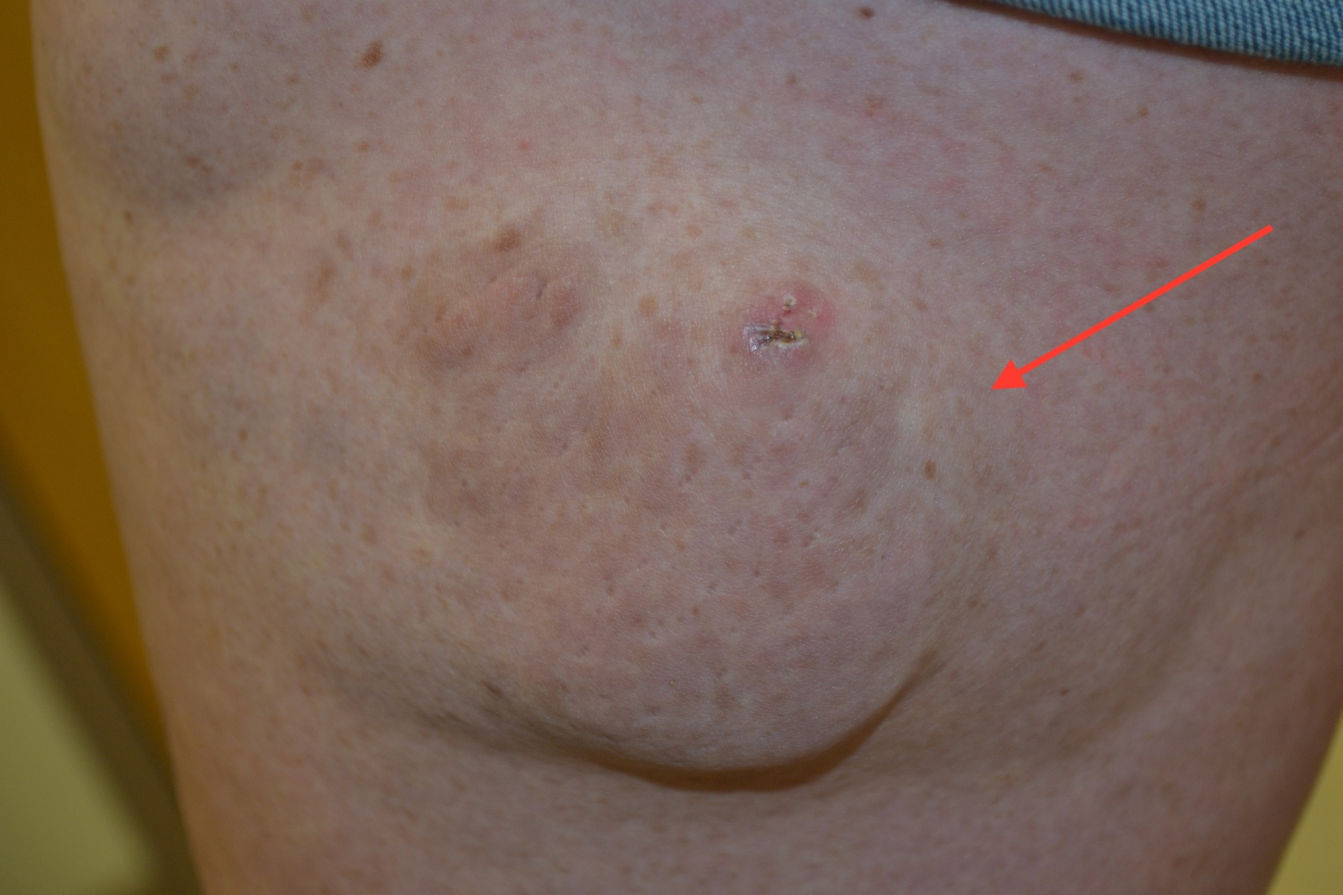
Plexiform Neurofibroma.

A 21-year-old Caucasian female gravida two parity one at 28 weeks presented to the dermatology clinic for evaluation of a large mass on her left lateral thigh (Figure 2). The patient had an existing diagnosis of NF One, but no other medical conditions. Denied any use of regular medication besides prenatal vitamins. The mass had been present since early childhood and had been stable in size since 11 to 12 years of age.

Per the patient, the mass started to grow early in her first trimester and was noted to be more painful than in the past. The patient reported pain with minor trauma that lasted for days. No change in consistency of the mass was noted. No reported weakness or altered sensation in the leg, night sweats, fevers, chills, or weight loss by the patient. In her past pregnancy, the patient denied any change to the size of the mass or increase in pain like she was currently experiencing.

The physical exam was notable for multiple 2-3 mm hyperpigmented macules in the bilateral axilla. The upper extremities and back had large hyperpigmented tan macules and patches in various sizes consistent with Cafe au lait spots. The left thigh had a large 18 x 9 cm boggy hyperpigmented mass on the lateral side. It was pendulous and had multinodular consistency. The mass was moderately tender on palpation. The patient was noted to be able to ambulate without difficulty and had equal sensation and strength in both lower extremities.

After discussion with the patient, she was prepped for punch biopsy of the mass. Multiple biopsies were taken from different areas of the mass to ensure adequate sampling. The results of the biopsy revealed plexiform neurofibroma without any indications of malignant changes in any of the sites. The patient returned to the clinic a week later for suture removal and was informed that the mass was a plexiform neurofibroma, but did not appear to be malignant at the time of biopsy.

The patient was educated about the risk of malignant transformation of the plexiform tumor and told to return if the mass changed in size again or became more painful. There were no indications of weakness in the extremity that would need to be evaluated further. The patient was advised to follow up with her obstetrician for routine pregnancy care. The patient was counseled to seek follow-up with an ophthalmologist for a full eye exam and to maintain regular follow-ups with her primary care physician.

## Discussion

Malignancy is a concern in the NF1 population as they are at a very high lifetime risk for the development of many types of cancer. There are some small studies that support tumor growth in pregnancy likely secondary to the increased level of hormones. Further, there has been documented rapid growth in preexisting tumors that have caused life-threatening and sometimes even fatal complications [3]. In a patient with an evolving tumor in the setting of known NF1, it must be considered as a possible malignancy until biopsy can be performed.

The patient described above had many clear diagnostic criteria for NF1 that were readily appreciable on physical exam. However, her most striking manifestation of NF1 and the reason for her presentation to the dermatology clinic was the plexiform lesion on her thigh. Per the patient, the plexiform neurofibroma had been slowly increasing in size during her pregnancy for many weeks before being to dermatology for evaluation.

The plexiform neurofibromas are an area of particular concern because they have the ability to undergo malignant degeneration into a malignant peripheral nerve sheath tumor (MPNST) [4]. The plexiform tumor is an area of ongoing research as they are resistant to radiation and chemotherapy. These tumors arise from multiple nerve fascicles and grow along the length of the nerve [1,5]. These tumors can be locally destructive and grow into surrounding tissue and bone. Typically, the plexiform tumors develop early in life and grow in the first decade then become relatively stable. During the period of growth, if the tumor becomes destructive, surgery is the best means of removing it from the patient. Although new treatments such as pegylated interferon – alpha – twoB or tyrosine kinase inhibitors are actively being investigated [1,5].

Plexiform neurofibromas are of particular concern because any change in their size after the first decade of life may be an indication of malignant degeneration into the MPNST. The patient described above had a large tumor on her thigh that had been stable since middle school and then in her first trimester it began to grow. Per the patient, the tumor also became painful when she experienced minor trauma like brushing up against a counter. New findings like increased size, consistency, pain, or new parenthesis should be promptly evaluated in any patient with NF1.

However, this patient was complicated by the fact that she was pregnant. Tumors have been documented to grow in the past during pregnancy. It is believed that increased amounts of progesterone and estrogen may play in the enlargement of tumors [4]. The evaluation of an enlarging plexiform tumor is by multiple biopsies to look for malignancy. Imaging like magnetic resonance imaging (MRI) has limited role as it cannot distinguish benign from malignant tumor. It is important to take multiple biopsies of the lesion to increase the probability of detecting an MPNST if it is present in the plexiform tumor. It is estimated that at least 80% of the MPNSTs arise from preexisting plexiform lesions [4,3]. The cumulative lifetime risk of a person with a plexiform tumor developing MPNST is 8-13% and these malignancies carry a poor prognosis with five-year survival rates around 16% [3].

## Conclusions

Early surgical intervention is key if MPNST is suspected in a patient with NF1. In rare circumstances where the patient is also pregnant it cannot be assumed that the tumor is enlarging secondary to hormones, as any delay in diagnosis would decrease the patient’s chance of survival. Malignancy must be considered in any women with a rapidly enlarging tumor. Malignancy is a large cause of morbidity and mortality in the NF1 population.
